# What’s Normal? Immune Profiling of Human Milk from Healthy Women Living in Different Geographical and Socioeconomic Settings

**DOI:** 10.3389/fimmu.2017.00696

**Published:** 2017-06-30

**Authors:** Lorena Ruiz, Irene Espinosa-Martos, Cristina García-Carral, Susana Manzano, Michelle K. McGuire, Courtney L. Meehan, Mark A. McGuire, Janet E. Williams, James Foster, Daniel W. Sellen, Elizabeth W. Kamau-Mbuthia, Egidioh W. Kamundia, Samwel Mbugua, Sophie E. Moore, Linda J. Kvist, Gloria E. Otoo, Kimberly A. Lackey, Katherine Flores, Rossina G. Pareja, Lars Bode, Juan M. Rodríguez

**Affiliations:** ^1^Department of Nutrition, Food Science and Food Technology, Complutense University of Madrid, Madrid, Spain; ^2^Probisearch S.L., C/Santiago Grisolía, Tres Cantos, Spain; ^3^School of Biological Sciences, Washington State University, Pullman, WA, United States; ^4^Paul G. Allen School for Global Animal Health, Washington State University, Pullman, WA, United States; ^5^Department of Anthropology, Washington State University, Pullman, WA, United States; ^6^Department of Animal and Veterinary Science, University of Idaho, Moscow, ID, United States; ^7^Department of Biological Sciences, University of Idaho, Moscow, ID, United States; ^8^Dalla Lana School of Public Health, University of Toronto, Toronto, ON, Canada; ^9^Department of Human Nutrition, Egerton University, Nakuru, Kenya; ^10^Division of Women’s Health, King’s College London, London, United Kingdom; ^11^MRC Unit, Serekunda, Gambia; ^12^Faculty of Medicine, Lund University, Lund, Sweden; ^13^Department of Nutrition and Food Science, University of Ghana, Accra, Ghana; ^14^Instituto de Investigación Nutricional, Lima, Peru; ^15^Department of Pediatrics, and Mother Milk Infant Center of Research Excellence (MoMICoRE), University of California, San Diego, La Jolla, CA, United States

**Keywords:** breastfeeding, human milk, lactation, immunoglobulins, cytokines, chemokines, growth factors

## Abstract

Human milk provides a very wide range of nutrients and bioactive components, including immune factors, human milk oligosaccharides, and a commensal microbiota. These factors are essential for interconnected processes including immunity programming and the development of a normal infant gastrointestinal microbiome. Newborn immune protection mostly relies on maternal immune factors provided through milk. However, studies dealing with an in-depth profiling of the different immune compounds present in human milk and with the assessment of their natural variation in healthy women from different populations are scarce. In this context, the objective of this work was the detection and quantification of a wide array of immune compounds, including innate immunity factors (IL1β, IL6, IL12, INFγ, TNFα), acquired immunity factors (IL2, IL4, IL10, IL13, IL17), chemokines (IL8, Groα, MCP1, MIP1β), growth factors [IL5, IL7, epidermal growth factor (EGF), granulocyte colony-stimulating factor, granulocyte–macrophage colony-stimulating factor, TGFβ2], and immunoglobulins (IgA, IgG, IgM), in milk produced by healthy women of different ethnicities living in different geographic, dietary, socioeconomic, and environmental settings. Among the analyzed factors, IgA, IgG, IgM, EGF, TGFβ2, IL7, IL8, Groα, and MIP1β were detected in all or most of the samples collected in each population and, therefore, this specific set of compounds might be considered as the “core” soluble immune factors in milk produced by healthy women worldwide. This approach may help define which immune factors are (or are not) common in milk produced by women living in various conditions, and to identify host, lifestyle, and environmental factors that affect the immunological composition of this complex biological fluid.

**Clinical Trial Registration:** www.ClinicalTrials.gov, identifier NCT02670278.

## Introduction

Human milk is uniquely suited to the infant’s nutritional needs and is also like a responsive and training substance that protects infants from a wide array of diseases in both developed and developing countries (1, 2). The presence of a wealth of bioactive factors in human milk—including cellular and soluble immune factors (3–5), human milk oligosaccharides (6), and live bacteria (7)—seems to be coordinately responsible for the unparalleled immunological, anti-inflammatory, and anti-infectious properties of this biological fluid. Bioactive components in human milk play key roles in the establishment of an efficient gastrointestinal (GI) barrier and a physiological GI microbiota in infancy, and in the training of the infant immune system, favoring the development of intestinal and systemic immune-homeostasis (8).

Throughout pregnancy, maternal B and T cells are selectively directed from blood and mucosal surfaces, including those of the GI and respiratory tracts, to the mammary gland, where they produce a wide range of immune factors essential to protect the inexperienced, mucosal-associated immune system of the newborn infant (9, 10). Therefore, the lactating mammary gland (and the colostrum and milk it produces) can be truly considered as a relevant part of the infant immune system where breastfeeding provides the postnatal link that promotes maternal–infant immune dialog (11). The effects of such fine programming are long-lasting and, in fact, breastfeeding has been associated to a significant reduction in the rates of allergic and respiratory diseases during adulthood (12–14).

Immunoglobulins (Ig) are the immune factors most studied in human milk. Dimeric IgA or pentameric IgM confer the infant immune protection against antigens to which the maternal mucosal-associated lymphoid tissues (MALTs) have been exposed and, therefore, to which the baby is very likely to be exposed during early life (11). IgA-coated bacteria can be detected in the infant GI tract, providing a mechanistic explanation for the IgA-mediated protection against neonatal infection and sepsis (15). Other immune factors present in human milk, including cytokines, chemokines, and growth factors [e.g., IL6, IL7, IL10, epidermal growth factor (EGF), TGFβ], contribute to differentiation of IgA-producing cells, playing a pivotal role in the maturation of the infant GI-associated immune system and in protecting the newborn against infectious diseases (16).

Maternal environmental factors, such as gestation length, birth mode, diet, time postpartum, or previous antigenic exposures are known to affect the immunological composition of human milk (17–19). Therefore, it is reasonable to assume that the concentrations of these substances in milk produced by healthy women may depend on an individual’s own life circumstances. Previous studies focused on the immunological composition of human milk have assessed a narrow panel of immune factors, have recruited women from a single location, and/or have included a relatively small sample size (20–26). In this context, the objective of this work was the assessment of a wide spectrum of immunological compounds, including innate immunity factors, acquired immunity factors, chemokines, growth factors, and Ig, in milk produced by healthy women of different ethnicities, living in high-, middle-, and low-income countries and, therefore, including very different geographical, dietary, socioeconomic, and environmental settings. International cohort studies, such as this, are fundamental in determining if there is a common set of “core” immune factors naturally present in human milk under various physiological conditions. In addition, studies such as this are needed to identify host, lifestyle, and environmental factors associated with (1) the presence/absence of and (2) variation in the concentration of these (and other) human milk-borne immunomodulatory constituents. Our overarching hypothesis was that “normal” varies in terms of immune components of human milk.

## Materials and Methods

### Experimental Design, Subjects, and Ethics Approvals

This investigation took place between May 2014 and April 2016 and was carried out as a cross-sectional, observational study involving eight contrasting countries. A total of 410 healthy breastfeeding women initially participated in the study, which was designed primarily to characterize global variation in the milk microbiome and oligosaccharide profiles. Results concerning the latter have been published previously (27). To be eligible for participation, women had to be breastfeeding or expressing milk at least five times daily (to assure adequate milk production); self-reported as healthy and nursing healthy infants; ≥18 years of age; and between 2 weeks and 5 months postpartum. Women did not need to be exclusively breastfeeding. Exclusion criteria included current indication of breast infection or breast pain that the woman did not consider “normal” for lactation; maternal use of antibiotics in the previous 30 days; or nursing a child with signs and/or symptoms of acute illness in the previous 7 days or who had taken antibiotics in the previous 30 days.

Our original sample included two European (Spanish and Swedish), one South American (Peruvian), two North American (USA), and six sub-Saharan African [rural and urban Ethiopian (ETU), rural and urban Gambian, Ghanaian (GN), and Kenyan] cohorts. Samples collected from rural Ethiopian women, however, were not analyzed in this work because they were initially preserved using a chemical preservative (rather than being frozen). Therefore, a total of 370 samples were included in the immunological analysis.

Spanish (SP) subjects (*n* = 41) were recruited in Madrid, Zaragoza, Huesca, and Vizcaya. Swedish (SW) subjects (*n* = 24) were recruited in or near Helsingborg and were self-reported as Nordic (both parents and all grandparents self-described as having only Swedish, Finnish, Danish, Icelandic, or Norwegian heritage). Peruvian (PE) subjects (*n* = 43) resided in a peri-urban area of Lima. The North American subjects were recruited in southeastern Washington and northwestern Idaho [USA/Washington (USW); *n* = 41] and southern California (USC; *n* = 19), the former being of unspecified ethnicity and the latter self-identified as Hispanic. ETU (*n* = 40) subjects self-identified as Sidama and resided in Hawassa, in the Southern Nations, Nationalities, and Peoples’ Region. Rural and urban Gambian (GBR and GBU, respectively) subjects self-identified as Mandinka. Urban Gambian participants (*n* = 40) were selected from the Bakauarea, while the rural cohort (*n* = 40) lived in the West Kiang region. Ghanaian subjects (*n* = 40) were Krobo or Dangme and lived in southeastern Ghana. Kenyan (KE) subjects (*n* = 42) were recruited from the multiethnic city of Nakuru.

Upon enrollment, each woman completed several questionnaires including one that ensured eligibility and another related to general maternal and infant health and anthropometry (Table 1). Data analyzed to examine possible influence from environmental, behavioral, and individual characteristics on immune composition of milk included the presence of animals in the home, birth mode, maternal and infant health problems and medication use, anthropometric measurements, dietary intake, maternal and infant age, and time since last feeding. Animals in the home referred to all pets and/or livestock that were kept, at least part of the day, within the home and included any and all types of animals (e.g., dogs and cats in the US and cattle in Ethiopia). Mothers also reported whether the focal infant was born vaginally or *via* cesarean section. The presence or absence of health problems and illnesses for the mother and focal infant in the postpartum period were collected through maternal reports. Mothers also recounted whether they received medication during the birth and whether they or the focal infants took medications in the postpartum period. Medication was widely defined, including Western pharmaceuticals and traditional, local remedies (e.g., medicinal teas). Our current analysis on dietary intake was limited to whether or not the mother was advised to consume fermented or cultured foods or beverages. Maternal and infant age was calculated to the nearest day. At some sites mothers did not know their or their infants’ birth dates. In such cases, maternal age was estimated based on the mother’s estimate with consideration given to her reproductive history (i.e., number of children both living and deceased). When an infant’s date of birth was not known, age was estimated based on the mother’s recollection and in some cases local events and conversations with nurses who knew the population. The precision of these estimates is likely to be excellent due to the age of the infants enrolled: little time had passed, thus decreasing the likelihood of error in self-reports. Body mass index (BMI) was calculated from mothers’ weights and heights. Time since last feeding was collected through maternal self-report. Mothers reported an estimated amount of time since the infant nursed on the breast of choice for sample collection.

**Table 1:** Main characteristics of the populations analyzed in the study.

Location	ETU	GBR	GBU	GN	KE	PE	SP	SW	USC	USW
Human development index classification*										
LHD	LHD	LHD	MHD	LHD	HHD	VHHD	VHHD	VHHD	VHHD

Animals in home	15	22	3	5	5	58	39	42	39	34

C-section	0	0	3	15	22	49	10	21	37	19

Infant health problems	5	54	50	28	22	7	22	4	44	32

Infant medication	5	46	57	37	88	21	17	17	47	50

Maternal health problems	12	37	43	8	20	5	22	17	17	31

Maternal postpartum medication	2	42	35	32	44	14	68	37	60	91
Maternal BMI**	^ab^	^ab^	^abc^	^acd^	^abcd^	^d^	^abc^	^abcd^	^d^	^cd^
Underweight	3	13	5	2	2	0	0	0	0	0
Normal weight	82	74	65	50	70	30	75	54	16	46
Overweight	15	13	25	35	20	33	15	25	42	30
Obese	0	0	5	13	8	37	10	21	42	24

Maternal age	^a^	^b^	^b^	^b^	^ab^	^ab^	^c^	^b^	^b^	^b^
≤24 years	85	45	36	25	52	46	2	12	16	24
24 < years ≤ 31	12	29	46	45	33	30	12	46	58	39
>31 years	3	26	18	30	14	23	85	42	26	37

Maternal medication before/during/post-delivery										
IPAb	5	5	15	22	33	33	34	21	21	32
No	67	50	45	50	41	33	22	37	5	12
Other	28	45	40	28	26	35	44	42	74	56

Time postpartum	^ab^	^a^	^ab^	^ab^	^ab^	^ab^	^ab^	^b^	^ab^	^a^
≤46 days	30	18	23	40	17	23	19	63	37	12
46 < days ≤ 63	28	28	27	15	21	30	24	25	21	27
63 < days ≤ 77	42	31	25	30	17	26	15	8	10	29
>77 days	0	23	25	15	45	21	42	4	32	32

Time since last feeding	^a^			^b^	^b^	^a^	^a^	^a^	^a^	^a^
<30 min	10	na	na	57	10	2	5	12	11	5
30 ≤ min < 60	20	na	na	35	90	19	35	4	11	3
60 ≤ min ≤ 120	28	na	na	5	0	63	35	46	50	10
>120 min	42	na	na	3	0	16	25	38	28	82

*Relative frequencies of each category, as calculated according to the information collected from the surveys, are represented for each geographical location*.

*ETU, urban Ethiopia; GBR, rural Gambia; GBU, urban Gambia; GN, Ghana; KE, Kenya; PE, Peru; SP, Spain; SW, Sweden; USC, USA/California; USW, USA/Washington; BMI: body mass index; IPAb, intrapartum antibiotherapy; na, not available*.

*Fischer χ^2^ test has been used for categorical and ranked variables; p < 0.05 was obtained for all the variables. For each ranked variable, different letters mean statistical differences in the pairwise post hoc comparison*.

*Human development index [United Nations Development Program (28)]: LHD, low human development; MHD, medium human development; HHD, high human development; VHHD, very-high human development.

**International classification of adult underweight, overweight, and obesity according to BMI [Global Database BMI, WHO; (93)]: underweight, BMI < 18.5; normal weight, 18.5 ≤ BMI < 25.0; overweight, 25 ≤ BMI < 30; and obese, BMI ≥ 30.0.

For each country, the human development indexes (HDI) from the United Nations Development Program (28) were also taken into consideration. HDI for each country was classified as low human development (LHD), which includes ETU, GBR, GBU, and KE; medium human development (MHD), which includes GN; high human development (HHD), which includes PE; or very-high human development (VHHD), which includes SP, SW, USC, and USW.

Ethics approvals were obtained for all procedures from each participating institution, with overarching approval from the Washington State University Institutional Review Board (#13264). After being translated from English (when needed), informed, verbal, or written consent (depending on locale and the subject’s literacy level) was acquired from each participating woman.

### Milk Collection and Preservation

Using gloved hands, research personnel or the mother (depending on cultural acceptability) cleaned the “study breast” (chosen by subject) twice with prepackaged castile soap towelettes (Professional Disposables International, Inc.; Orangeburg, NY, USA) using a newly opened package each time. When deemed appropriate, this step was preceded by a general cleansing with water (and soap if needed) to remove noticeable soil. In PE, SW, USC, and USW cohorts, at least 20 mL (typically 40–60 mL) of milk samples were then collected into a single-use, sterile polypropylene milk collection container with a polybutylene terephthalate cap (Medela, Inc.; McHenry, IL, USA) using an electric breast pump. In the remaining sites, ~20 mL of milk were collected. In SP, milk samples were collected *via* manual expression (using a gloved hand) into single-use, sterile polypropylene milk collection containers with polybutylene terephthalate caps (Medela, Inc.; McHenry, IL, USA). In the remaining sites, milk was manually expressed (using a gloved hand) into sterile polypropylene specimen containers with polyethylene caps (VWR International, LLC.; Visalia, CA, USA). To help control for known and unknown biases that might be introduced by using different materials, all milk collection supplies (gloves, wipes, collection containers, etc.) were standardized and provided to study personnel at each site.

Milk was immediately placed in ice or in a cold box (4°C) where it remained until it was partitioned, within 1 h, into aliquots. Milk was immediately frozen (−20°C) and, then, transferred to −80°C for long-term storage. Samples collected outside from Madrid were shipped on dry ice (−78.5°C) to the Complutense University of Madrid where all the immunological determinations were performed. In order to eliminate or minimize potential lab biases, all the samples were submitted to a single freeze–thaw cycle and were analyzed by the same researchers using the same reagents’ batches and equipment.

### Immunological Analysis

The concentrations of innate immune factors (IL1β, IL6, IL12, IFNγ, TNFα), acquired immunity factors (IL2, IL4, IL10, IL13, IL17), chemokines (IL8, Groα, MCP1, MIP1β), and growth factors [IL5, IL7, granulocyte colony-stimulating factor (GCSF), granulocyte–macrophage colony-stimulating factor (GMCSF), TGFβ2] were determined by magnetic bead-based multiplex immunoassays, using a Bioplex 200 instrument (Bio-Rad, Hercules, CA, USA) and the Bio-PlexPro Human Cytokine, Chemokine, and Growth Factor Assays (Bio-Rad), according to manufacturer’s instructions. TGFβ2 was acid activated prior to the analysis as recommended by the manufacturer. EGF was determined by ELISA using the RayBio Human EGF ELISA kit (RayBiotech, Norcross, GA, USA). Concentrations of Ig (IgA, total IgG, and IgM) were determined using the Bio-Plex Pro Human Isotyping Assay kit (Bio-Rad) in the Bioplex system instrument.

Prior to their analysis, samples (1 mL) were processed and aliquoted as described previously (29). A fresh aliquot was used for each assay, avoiding defrosting cycles. Every assay was run in duplicate according to manufacturer’s instructions, and standard curves were performed for each analyte on every assay. Cytokine concentrations were expressed as nanograms per liter, Ig concentrations as milligrams per liter, and concentrations of EGF, TGFβ2, and Groα as micrograms per liter. The inter-assay coefficients of variation were below manufacturers’ instructions for all the immune markers, and the detection limits of the assays are shown in Table S1 in Supplementary Material.

### Statistical Analysis

Normality of data distribution was interrogated through visual inspection of histograms and Shapiro–Wilk test, both evidencing non-normal distribution for all tested variables (*p* < 0.05). Accordingly, non-parametric statistical analyses were used. Differences in recorded demographic data and detection frequencies of the immunological compounds were evaluated among locations by Fisher test followed by a *post hoc* Nemenyi test adjusted to χ^2^ statistics for pairwise multiple comparisons. Descriptive univariate analysis was performed comparing the concentrations of all parameters analyzed for the 10 different subpopulation groups through unadjusted Kruskal–Wallis test and further *post hoc* Nemenyi test for pairwise multiple comparisons. For each pairwise combination of the immunological compounds analyzed, the Sørensen–Dice index was calculated, and a co-occurrence matrix was constructed. A heatmap representing the values of co-occurrence indexes was then plotted. To summarize the results of the immunological profiles, exploratory multivariate analyses, such as principal component analysis (PCA) with a variable reduction approach (cos^2^ > 0.2), were performed using the R package *FactoMineR*.

Agglomerative hierarchical clustering, using the Euclidean distance and Ward methods (R package: dendextend), was used to study the binary matrix of detection and the measured amount matrix of immune factors included in the study. Subsequently, a heatmap representing the detection of the immune factors with the sample labels replaced by a colored bars vector for HDI classification was plotted. The dendrogram obtained for measured amounts of immune factors was also represented as a circularized tree of the samples colored by location. To investigate potential associations between the immunological variables and the categorical variables describing demographic aspects of the populations, generalized linear models (GLMs) were performed. Significance was declared at *p* < 0.05 for all analyses. All analyses were performed with the R software version 3.3.2 (R-project, http://www.r-project.org).

## Results

### Analysis of Maternal Health, Infant Health, Lifestyle, and Anthropometry Data

Maternal health, infant health, lifestyle, and anthropometric parameters that were analyzed in this study are shown in Table 1. Analysis of the data by the Friedman rank sum test revealed significant differences for all the parameters among all the populations. Notable differences included (a) maternal age, which was the highest in the SP cohort (median: 34.0 years) and the lowest in ETU (median: 20.5 years); (b) postpartum days at the time of sample collection, a period that was the shortest in SW (median: 42 days) and the longest in KE (median: 74 days); and (c) C-section rates, which ranged from 48.8% in Peru to 0% in ETU and GBR. Globally, infant medication rate was highest in KE (88.5%), while maternal medication was more frequent among USW, where 56.1% of the mothers received medication (different from intrapartum antibiotic therapy) during pregnancy or delivery, and 91% of them declared that they had received medication during the postpartum period. ETU and PE mothers presented the lowest rates of postpartum medication as 97.5 and 86.0% of subjects, respectively, reported no postpartum medication.

### Frequency of Detection of the Immunological Compounds in the Milk Samples

**Figure 1:**
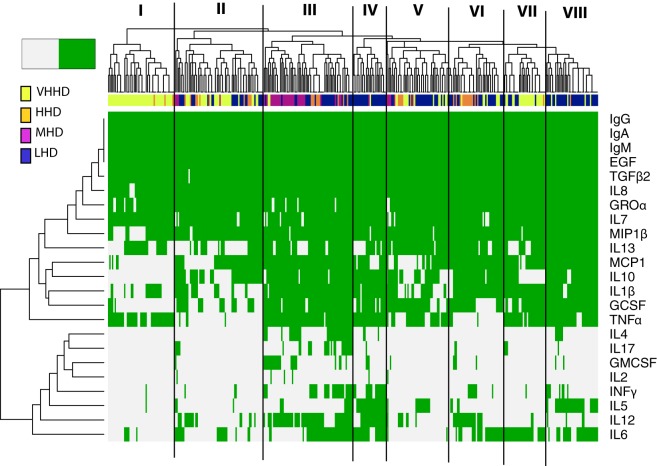
Heatmap representing the detection frequency of the immunoglobulins, cytokines, growth factors, and chemokines quantified in human milk (green: present; grey: absent). Each row represents the detection frequency for an individual specific immune factor as indicated on the *y*-axis, and each column represents an individual sample/subject. Hierarchical clustering of the 365 samples analyzed is shown in the upper dendrogram. Color-coded bar accompanying the dendrogram represents the level of development of the geographical locations analyzed, according to the human development index [United Nations Development Program; (28)]. LHD, countries with low human development index; MHD, countries with medium human development index; HHD, countries with high human development index; VHHD, countries with very-high human development index.

All the immunological factors could be detected among at least some of the human milk samples analyzed in this study, although at highly variable frequencies and concentrations. Globally, IgA and EGF displayed the highest frequencies of detection (100% of the samples), followed by IgG, IgM, TGFβ2, IL7, IL8, and Groα, which were detected in most of the samples collected from each population (Table 2; Figure 1). The detection frequency of MIP1β was high (>91%) in all populations with the exception of samples from USW (51%). IL1β, TNFα, GCSF, IL6, IL13, and MCP1 were also detected in all the populations, but their frequencies varied depending on the group. Some immune compounds exhibited intermediate frequencies of detection in certain locations but could not be detected among samples collected elsewhere. They included IL2 (detected exclusively in some GN samples), IL4 (not detected in USW, SW, and GBU), IL10 (not detected in SW, USC, and USW), IL17 (not detected in SW and USW), IL5 (not detected in GBU, SW, and USW), IL12 (not detected in USW), and INFγ (not detected in USW). Finally, low frequencies of detection were found for GMCSF, which was detected in less than 10% of the samples within each group; except GN where it was detected in 50% of the samples; and with the exceptions of SW, USC, and USW where this factor could not be detected in any sample. IL2, IL17, and IL4 were also found in very low frequencies: <18, 22, and 33%, respectively, across all locations.

**Table 2:** Relative frequencies of detection of each immune factor in human milk within each population.

	ETU (*N* = 40)	GBR (*N* = 40)	GBU (*N* = 40)	GN (*N* = 40)	KE (*N* = 42)	PE (*N* = 43)	SP (*N* = 41)	SW (*N* = 24)	USC (*N* = 19)	USW (*N* = 41)	*p*-Value*
IL1β	73^a^	83^a^	70^a^	98^a^	98^a^	72^a^	56^ab^	58^ab^	84^a^	24^b^	<0.001
IL6	35^ab^	75^a^	50^ab^	33^ab^	69^a^	42^ab^	41^ab^	33^ab^	47^ab^	19^b^	<0.001
IL12	13^a^	68^bc^	30^ab^	75^c^	24^a^	74^c^	17^a^	13^a^	10^a^	0^a^	<0.001
INFγ	25^ab^	40^a^	18^ab^	18^ab^	26^ab^	26^ab^	2^b^	8^ab^	10^ab^	0^b^	<0.001
TNFα	100^a^	83^ab^	30^c^	85^ab^	62^abc^	39^bc^	44^bc^	67^abc^	79^abc^	76^abc^	<0.001
IL2	0	0	0	18	0	0	0	0	0	0	
IL4	33^a^	23^ab^	0^bc^	33^a^	14^abc^	7^bc^	2^bc^	0^c^	5^bc^	0^c^	<0.001
IL10	63^a^	98^a^	95^a^	90^a^	100^a^	79^a^	78^a^	0^b^	0^b^	0^b^	<0.001
IL13	100^a^	68^ab^	45^b^	68^ab^	67^ab^	86^ab^	83^ab^	50^b^	53^ab^	66^b^	<0.001
IL17	10	15	8	18	21	9	5	0	5	0	0.022
IL5	43^abd^	65^bd^	0^c^	15^ac^	60^bd^	5^c^	7^ac^	8^ac^	0^c^	0^c^	<0.001
IL7	100^a^	100^a^	93^a^	73^b^	100^a^	100^a^	93^a^	100^a^	100^a^	100^a^	<0.001
IgA	100	100	100	100	100	100	100	100	100	100	1
IgM	100	100	100	100	100	98	100	96	100	100	1
IgG	100	100	100	100	100	98	100	96	100	100	1
TGFβ2	100	100	100	100	100	100	100	96	100	100	0.114
IL8	100^a^	100^a^	100^a^	100^a^	100^a^	100^a^	100^a^	100^a^	100^a^	90^b^	<0.001
GROα	100^a^	100^a^	100^a^	80^b^	100^a^	100^a^	100^a^	100^a^	100^a^	88^ab^	<0.001
MCP1	85^ab^	85^ab^	83^ab^	95^a^	90^ab^	65^ab^	78^ab^	46^bc^	58^abc^	17^c^	<0.001
MIP1β	100^a^	100^a^	98^a^	98^a^	100^a^	98^a^	95^a^	92^a^	95^a^	51^b^	<0.001
GCSF	93^a^	73^a^	25^bc^	98^a^	90^a^	79^a^	71^a^	50^abc^	63^ab^	10^c^	<0.001
GMCSF	10^a^	3^a^	5^a^	50^b^	5^a^	5^a^	7^a^	0^a^	0^a^	0^a^	<0.001
EGF	100	100	100	100	100	100	100	100	100	100	1

*ETU, urban Ethiopia; GBR, rural Gambia; GBU, urban Gambia; GN, Ghana; KE, Kenya; PE, Peru; SP, Spain; SW, Sweden; USC, USA/California; USW, USA/Washington; EGF, epidermal growth factor; GCSF, granulocyte colony-stimulating factor; GMCSF, granulocyte–macrophage colony-stimulating factor*.

*Kruskal–Wallis test. Different caption letters mean statistical differences when the post hoc pairwise comparison Nemenyi test was done.

### Quantification of the Immunological Compounds in the Milk Samples

**Figure 2:**
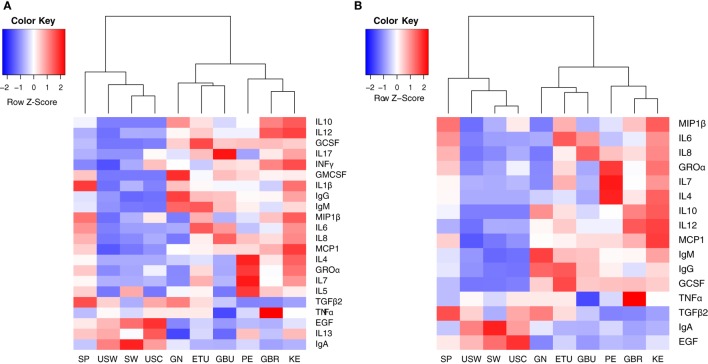
Heatmaps representing the median concentrations of different immune factors in each location where the samples were collected from. **(A)** Heatmap representing the median concentrations of all the immune factors assayed in this study. **(B)** Heatmap representing the median concentrations of the 16 immune factors that contributed the most to samples separation according to the multivariate analysis conducted in this work (cos^2^ > 0.2). SP, Spain; USW, USA/Washington; SW, Sweden; USC, USA/California; GN, Ghana; ETU, urban Ethiopia; GBU, urban Gambia; PE, Peru; GBR, rural Gambia; KE, Kenya.

Median values of all the immune factors analyzed in this study are recorded in Tables 3–5 and are also summarized as a heatmap in Figure 2A, as described below.

**Table 3:** Median concentration (in nanograms per liter) and interquartile ranges for the factors in human milk associated to innate immunity.

Location	IL1β	IL6	IL12	INFγ	TNFα
ETU	0.57 (0.25–0.87)^abc^	15.37 (1.33–62.86)^ab^	2.61 (0.64–22,07)^ab^	10.93 (6.20–25.18)	4.67 (3.39–7.00)^ab^
GBR	0.40 (0.28–1.08)^abc^	7.06 (3.56–16.10)^ab^	4.16 (2.82–8.11)^a^	26.57 (18.35–38.66)	8.63 (5.23–13.50)^b^
GBU	0.52 (0.24–3.43)^ab^	12.51 (4.14–31.86)^ab^	1.71 (1.23–4.23)^ab^	19.25 (12.52–44.75)	1.27 (0.91–2.32)^a^
GN	0.74 (0.39–1.95)^a^	4.03 (1.10–16.23)^ab^	2.04 (1.20–3.03)^ab^	15.52 (1.14–116.47)	4.67 (3.68–7.46)^ab^
KE	0.98 (0.61–1.70)^a^	12.85 (5.80–43.19)^a^	4.46 (1.85–5.79)^a^	31.84 (21.16–82.65)	6.83 (2.08–12.47)^ab^
PE	0.52 (0.24–1.15)^abc^	5.11 (1.83–19.52)^ab^	1.71 (1.23–2.97)^ab^	18.33 (9.94–53.78)	3.65 (1.26–12.68)^ab^
SP	1.14 (0.27–2.61)^ab^	12.69 (6.13–19.21)^a^	0.86 (0.12–0.96)^b^	4.70 (4.70–4.70)	3.18 (1.76–4.98)^a^
SW	0.32 (0.26–1.21)^abc^	3.48 (2.81–5.39)^ab^	0.78 (0.61–37.71)^ab^	8.06 (1.28–14.85)	3.65 (1.50–5.54)^a^
USW	0.17 (0.11–0.58)^bc^	3.61 (1.07–10.33)^ab^	0.77 (0.61–0.94)^ab^	21.35 (2.16–40.55)	4.98 (2.83–7.07)^ab^
USC	0.12 (0.06–0.25)^c^	2.13 (0.18–4.94)^b^	nd	nd	4.45 (1.72–7.07)^a^

*p*-Value*	<0.001	0.003	<0.001	0.258	<0.001

*Results are expressed as median (IQR). nd, below detection limit. ETU, urban Ethiopia; GBR, rural Gambia; GBU, urban Gambia; GN, Ghana; KE, Kenya; PE, Peru; SP, Spain; SW, Sweden; USC, USA/California; USW, USA/Washington*.

*Kruskal–Wallis test. Different caption letters mean statistical differences when the post hoc pairwise comparison Nemenyi test was done.

**Table 4:** Median and interquartile ranges for the measured concentrations of factors in human milk associated to acquired immunity.

Location	IL2	IL4	IL10	IL13	IL17	IL5	IL7	IgA	IgM	IgG	TGFβ2
ETU	nd	0.41 (0.11–0.84)^ab^	4.44 (2.55–6.67)^ab^	1.77 (1.34–3.15)^ac^	25.55 (8.42–46.17)	2.05 (1.18–2.44)	52.54 (17.17–88.17)^ad^	323.22 (223.37–469.52)^ad^	83.93 (45.36–120.48)^a^	96.09 (72.22–127.69)^abc^	1.38 (0.43–3.29)^abc^
GBR	nd	0.83 (0.54–2.26)^ab^	7.07 (4.80–8.23)^ac^	1.57 (0.86–2.95)^ac^	19.23 (6.42–98.50)	2.69 (1.79–3.80)	32.14 (16.87–55.08)^ade^	235.78 (159.56–334.94)^a^	37.06 (22.82–82.37)^ab^	74.73 (44.04–116.76)^ac^	0.71 (0.28–1.65)^a^
GBU	nd	nd	2.50 (1.53–3.78)^b^	0.78 (0.39–2.15)^ab^	50.28 (5.54–1,048.68)	nd	13.33 (4.97–24.16)^b^	312.22 (215.57–510.98)^acd^	59.45 (29.30–121.46)^ab^	93.47 (67.53–150.23)^abc^	0.99 (0.26–2.40)^ab^
GN	5.62 (1.25–101.86)	0.27 (0.15–0.41)^a^	6.68 (4.44–8.10)^ac^	0.45 (0.28–0.61)^b^	11.66 (10.49–20.73)	1.77 (0.26–3.18)	2.39 (1.35–5.42)^c^	584.75 (377.08–994.91)^be^	80.87 (51.06–168.98)^a^	142.37 (81.16–214.73)^b^	1.78 (1.09–3.41)^bc^
KE	nd	3.43 (0.96–5.38)^b^	8.16 (7.01–9.33)^c^	2.73 (1.73–3.93)^c^	29.33 (9.87–68.59)	2.16 (1.39–3.18)	58.99 (19.86–106.59)^ad^	316.81 (218.24–463.82)^ad^	53.70 (36.56–85.45)^ab^	106.19 (76.94–174.81)^ab^	0.82 (0.46–3.21)^abc^
PE	nd	2.34 (0.19–4.82)^ab^	2.34 (0.19–4.82)^b^	2.67 (1.71–5.82)^c^	2.64 (1.07–46.59)	4.70 (0.43–8.97)	91.61 (36.71–131.55)^d^	499.57 (418.76–642.70)^bcf^	48.37 (33.59–68.67)^ab^	73.44 (57.54–100.41)^abc^	0.70 (0.41–1.21)^a^
SP	nd	0.70 (0.70–0.70)^ab^	3.25 (1.68–4.35)^b^	2.63 (1.48–3.99)^c^	4.29 (2.10–6.47)	2.57 (0.79–2.82)	34.56 (26.81–53.67)^ade^	418.83 (256.78–539.24)^bcd^	38.80 (19.92–62.45)^bd^	59.95 (48.73–90.51)^c^	1.99 (1.07–3.57)^c^
SW	nd	nd	nd	2.06 (1.23–3.68)^ac^	nd	3.09 (2.45–3.73)	11.15 (8.48–26.39)^bce^	1,840.18 (1,065.84–2,435.49)^e^	13.54 (4.13–17.65)^c^	15.31 (13.88–19.45)^d^	0.88 (0.53–1.78)^ab^
USC	nd	1.89 (1.89–1.89)	nd	3.59 (1.34–4.99)^ac^	16.84 (16.84–16.84)	nd	13.92 (7.23–31.56)^ab^	1,210.59 (642.90–2,053.42)^ef^	12.27 (8.98–18.91)^cd^	19.26 (13.89–36.37)^d^	1.60 (1.00–2.40)^abc^
USW	nd	nd	nd	2.87 (1.67–6.60)^c^	nd	nd	12.26 (8.70–15.54)^bc^	1,355.60 (849.41–2,112.45)^e^	18.95 (7.78–36.60)^cd^	32.67 (19.35–44.60)^d^	1.43 (0.85–2.29)^abc^

*p*-value*	NA	0.007	<0.001	<0.001	0.440	0.281	<0.001	<0.001	<0.001	<0.001	<0.001

*Concentrations of cytokines are expressed as nanograms per liter; concentrations of immunoglobulins as milligrams per liter; and concentration of TGFβ2 as micrograms per liter*.

*Results are expressed as median (IQR). nd, below detection limit*.

*ETU, urban Ethiopia; GBR, rural Gambia; GBU, urban Gambia; GN, Ghana; KE, Kenya; PE, Peru; SP, Spain; SW, Sweden; USC, USA/California; USW, USA/Washington*.

*Kruskal–Wallis test. Different caption letters mean statistical differences when the post hoc pairwise comparison Nemenyi test was done.

**Table 5:** Median and interquartile ranges for the measured concentrations of chemokines and growth factors in human milk.

Location	IL8	Chemokines			Growth factors		
		GROα	MCP1	MIP1β	GCSF	GMCSF	EGF
ETU	54.01 (27.55–152.11)^ad^	5.63 (3.14–11.75)^ad^	132.62 (49.71–338.41)^ab^	24.75 (14.53–57.33)^ad^	79.91 (43.77–134.61)^a^	8.63 (2.80–12.16)	4.65 (3.63–5.70)^ac^
GBR	59.56 (44.44–167.65)^a^	4.19 (1.72–8.05)^ab^	175.50 (70.43–612.74)^ab^	23.01 (11.46–47.96)^ab^	51.38 (32.08–99.01)^ab^	9.99 (9.99–9.99)	3.97 (3.51–5.24)^ab^
GBU	98.52 (36.06–278.61)^a^	1.36 (0.71–3.98)^bc^	147.31 (69.24–465.11)^ab^	9.48 (5.50–16.84)^bc^	48.19 (24.53–248.00)^ab^	13.44 (2.33–24.55)	3.53 (2.78–4.95)^ab^
GN	6.74 (2.29–17.36)^b^	0.27 (0.05–0.74)^c^	126.00 (45.79–260.11)^ab^	4.24 (2.71–9.15)^ce^	47.10 (33.76–63.58)^ab^	23.36 (8.18–42.91)	3.20 (2.21–4.11)^b^
KE	85.62 (59.15–228.34)^a^	8.96 (3.00–13.75)^a^	252.51 (109.92–807.72)^a^	33.11 (19.38–75.79)^a^	47.32 (17.45–87.68)^ab^	10.39 (9.58–11.21)	4.95 (4.10–6.12)^ac^
PE	67.90 (30.30–143.28)^ac^	11.01 (3.78–15.02)^ab^	148.70 (66.41–308.81)^ab^	14.24 (6.74–28.35)^bd^	50.20 (13.72–125.33)^ab^	11.84 (1.79–21.89)	4.19 (3.68–4.62)^ab^
SP	72.08 (27.73–183.59)^ac^	6.19 (3.61–10.15)^ad^	156.57 (59.10–307.49)^ab^	30.70 (15.17–74.08)^ad^	18.33 (4.88–59.06)^bd^	12.87 (0.44–23.83)	5.96 (4.73–6.85)^cd^
SW	11.66 (4.12–23.24)^b^	1.31 (0.54–5.71)^bde^	35.18 (18.60–231.82)^b^	9.42 (3.01–24.23)^bc^	1.29 (0.88–2.97)^c^	nd	8.29 (6.12–10.78)^d^
USC	22.30 (10.94–27.20)^bcd^	3.82 (0.95–7.19)^ab^	52.60 (24.19–142.64)^ab^	19.12 (3.41–31.09)^abd^	3.13 (1.94–6.51)^cd^	nd	9.42 (6.22–10.55)^d^
USW	5.19 (2.57–12.19)^b^	0.34 (0.21–0.73)^ce^	14.31 (12.33–127.92)^b^	2.85 (1.37–7.68)^e^	0.85 (0.18–1.36)^c^	nd	6.85 (5.76–8.51)^d^

*p*-value*	<0.001	<0.001	<0.001	<0.001	<0.001	0.496	<0.001

*Concentrations of GROα and EGF are expressed as micrograms per liter and the concentrations of other chemokines and growth factors as nanograms per liter*.

*Results are expressed as median (IQR). nd, below detection limit*.

*ETU, urban Ethiopia; GBR, rural Gambia; GBU, urban Gambia; GN, Ghana; KE, Kenya; PE, Peru; SP, Spain; SW, Sweden; USC, USA/California; USW, USA/Washington; EGF, epidermal growth factor; GCSF, granulocyte colony-stimulating factor; GMCSF, granulocyte–macrophage colony-stimulating factor*.

*Kruskal–Wallis test. Different caption letters mean statistical differences when the post hoc pairwise comparison Nemenyi test was done.

#### Innate Immunity Factors

The concentrations of the innate immunity factors IL1β, IL6, IL12, and TNFα displayed significant differences across geographical locations, in contrast to those of INFγ, which was relatively consistent across cohorts (Table 3). Although IL1β displayed the lowest values in all locations, significant differences were detected among groups: SP samples showed the highest (median: 1.14 ng/L; range: 0.27–2.61 ng/L) and USC the lowest (median: 0.12 ng/L; range: 0.06–0.25 ng/L) concentrations. The lowest IL6 and IL12 concentration values were recorded in USC, USW, and SW, where median concentrations ranged from 2.13 to 3.61 ng/L for IL6, and from below detection to 0.86 ng/L for IL12. On the contrary, the highest concentrations of these two factors were detected in ETU, KE, GBU, and SP for IL6 (range: 12.51–15.37 ng/L) and in KE and GBR for IL12 (range: 4.16–4.46 ng/L). The lowest values of TNFα were detected in GBU (median: 1.27 ng/L; range: 0.91–2.32 ng/L) and the highest in GBR (median: 8.63 ng/L; range: 5.23–13.50 ng/L), followed by KE (median: 6.83 ng/L; range: 2.08–12.47 ng/L). All the other locations showed intermediate TNFα levels ranging from 3.18 to 4.98 ng/L (Table 3; Figure 2A).

#### Acquired Immunity Factors

The highest IgA concentrations were found in SW, USC, and USW samples with median concentrations ranging from 1,210 to 1,840 mg/L. Interestingly, samples from these same locations contained the lowest IgG and IgM concentrations, ranging from 15.31 to 32.37 mg/L and from 12.27 to 18.95 mg/L, respectively (Table 4). Among the other factors related to acquired immunity, concentrations of IL4, IL10, IL13, and TGFβ2 were different across locations, but no clear patterns were observed except that once again, in SW, USC, and USW concentrations of IL10 were below the assay detection limit (Table 4). Concentrations of IL7 were lower in SW, USC, USW, and GBU (range: 11.10–13.92 ng/L) when compared to other locations (range: 32.14–91.61 ng/L), with the exception of GN (median: 2.39 ng/L). In relation to this location, the levels of acquired immunological factors in GN samples were quite different when compared to those exhibited by the other African locations: GN samples had the lowest IL13 and IL7 concentrations and the highest IgA and IgG concentrations among the African samples (Table 4). It must also be highlighted that, among all the samples analyzed in this study, IL2 was only detected in seven GN samples. No differences were found in IL5 and IL17 concentrations among the locations where these two factors were above the detection limits of the assay (Table 4; Figures 2A,B).

#### Chemokines

Groα was the most abundant chemokine in the milk samples analyzed in this study and, in fact, its levels were between 100- and 500-fold greater than those obtained for the rest of chemokines (Table 5; Figures 2A,B). Concentrations of all chemokines were significantly different across geographical locations (Table 5). IL8 exhibited the highest concentrations (54–98 ng/L) in SP, PE, and in the African locations with the exception, again, of GN (~7 ng/L). Median IL8 levels in SW, USC, and USW ranged from 5 to 22 ng/L. MCP1 concentrations were also lower in SW, USC, and USW (range: 14.32–52.60 ng/L), as compared to the other locations (126–252 ng/L). No clear patterns were observed in the distribution of Groα and MIP1β concentrations across the locations. Globally, KE samples displayed higher chemokine concentrations than those from other study sites.

#### Growth Factors

In relation to growth factors, no significant differences were found for GMCSF concentrations while GCSF and EGF showed significant variation depending on the location (Table 5; Figures 2A,B). Interestingly, GCSF and EGF showed opposite trends (lower GCSF concentrations, higher EGF concentrations) in the samples from VHHD locations (SP, SW, USC, and USW).

### Multivariate Analysis

The detection frequencies of the immune compounds were further evaluated by clustering analysis and heatmap plotting (Figure 1). Globally, these analyses suggest that the immune profiles of milk samples from healthy breastfeeding women can be, at least in part, differentiated according to the geographic origin of the samples’ donors. At a linkage distance of three, hierarchical clustering of detection frequencies of the immune factors showed eight different clusters, showing a high consistency with the HDI of the countries where samples were obtained. Some clusters were highly enriched in individuals from a specific geographical location, as it was the case of clusters I, II, and VII, which mainly encompassed samples from VHHD locations (SP, SW, USW, and USC); most of the samples from the MHD location (GN) are included in cluster III, which also contained some samples from LHD (ETU, GBR, GBU, and KE) and HHD (PE) locations. Clusters IV and VIII mostly comprised samples from LHD locations, and clusters V and VI were heterogeneous, including samples from LHD, HHD, and VHHD locations.

IgA, IgG, IgM, TGFβ2, EGF, IL7, IL8, and Groα formed a “core” set of immune factors that were detectable in all or most of the samples analyzed in this work, independent of the location where the samples were collected. Among the immune factors that allowed differentiation of samples in clusters, IL10 and/or IL13 were frequently absent in clusters enriched in samples from HHD locations (I, II, and VII). MIP1β was absent in most samples from cluster I, encompassing a great proportion of the samples from VHD locations. Likewise, IL4, IL17, and GMCSF detection was mostly limited to some samples from cluster III. In addition, the vast majority of samples from LHD and MHD locations (such as those grouping together in clusters III, IV, and VIII) were frequently characterized by detection of MCP1, IL10, IL1β, GCSF, and TNFα. In addition, GMCSF was detectable in many GN samples (MHD), whereas its detection in other samples was limited. Remarkably, the number of immune factors with concentrations below the detection limits was higher in samples from more highly developed locations (median: 11) when compared to those collected in regions with lower development (median 7) (Kruskal–Wallis, *p* < 0.05). In addition, four of the factors determined in this study (IL10, IL5, IL12, and INFγ) could only be detected in samples from LHD locations and were not detected in any from the highly developed ones. Concentration profiles of the immune factors studied also clustered with location and HDI classification of the location where samples were collected from (Figures S1–S3 in Supplementary Material).

**Figure 3:**
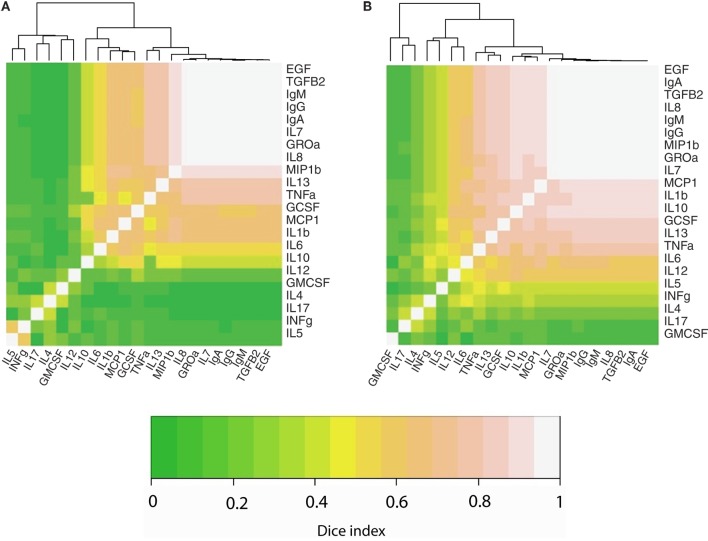
Heatmaps representing the co-occurrence frequency among immune factors analyzed in human milk, as determined per Sorensen–dice index in developed **(A)** and developing **(B)** countries. Lighter colors indicate strongest co-occurrence frequency.

We also performed an analysis to determine the co-occurrence profiles among the 23 immune factors evaluated in this study. For this purpose, the Sørensen–Dice similarity index was calculated individually for developed (USC, USW, SW, SP, PE) and developing (GBR, GBU, GN, KE, ETU) countries; these results are illustrated in a heatmap (Figures 3A,B). Hierarchical clustering evidenced three different clusters (a high co-occurrence cluster, a medium co-occurrence cluster, and a low co-occurrence cluster) in the two analyzed settings. However, the association pattern of immune factors belonging to each cluster was different among locations. Remarkably, the number of immune factors displaying a high co-occurrence was higher in developing locations (*n* = 15) as opposed to samples from developed settings (*n* = 11). As an example, while Ig, EGF, and TGFβ2 displayed high co-occurrence in both settings, the high co-occurrence cluster included also MCP1, IL1β, IL10, GCSF, and TNFα in developing locations. In addition, the number of low co-occurrence factors samples was lower in developing locations (*n* = 3) than in developed locations (*n* = 6). In this context, some of the immune factors that were predominantly detected in developing locations (e.g., IL5 or INFγ) exhibited medium co-occurrence in such locations but low co-occurrence in the developed regions.

**Figure 4:**
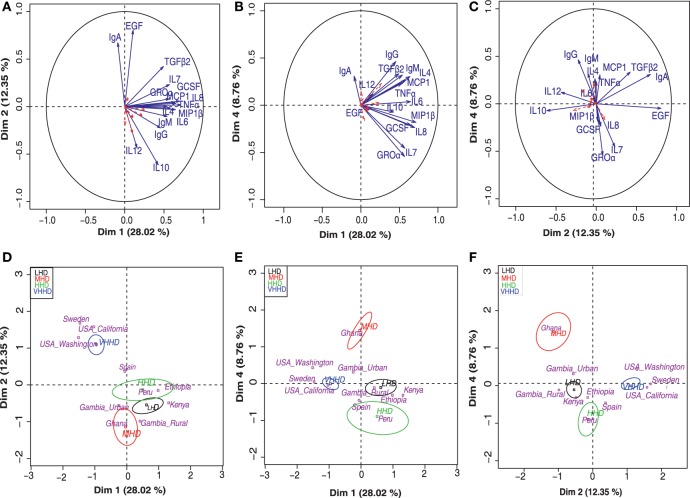
Principal component analysis (PCA) for the concentration of various immune factors in human milk. Top panels **(A–C)** represent separation into bidimensional maps, each one representing different dimension combinations. Arrows indicate variable factors contributing the most to the separation of the samples along the map. Variables with a cos^2^ > 0.2 are represented in blue and those with a lesser contribution to the graph are indicated in red dashed arrows. Lower panels **(D–F)** represent the distribution of the samples’ centroids of the different geographical locations (pink letters) along the same dimension combinations displayed in the respective upper panels **(A–C)**. Ellipsoids include at least 50% of the individuals belonging to each of the represented categories, which include VHHD, HHD, MHD, and LHD countries [United Nations Development Program; (28)].

Principal component analysis-based clustering of the detected concentrations of immunological factors was performed to summarize and discriminate sample subgroups based on their immune profiles. A total of five independent components with eigen value >1 globally explained 66% of the observed variability. This analysis showed that the immune factors IL1β, IL2, IL5, IL13, IL17, GMCSF, and INFγ exhibited a cos^2^ < 0.2 and, thus, had little or no contribution to the samples’ positioning along the bidimensional map (they are represented as dashed red arrows in the factor maps). On the other hand, among the variables contributing the most to samples’ positioning in the bidimensional map, high IgA levels seemed to be driving the separation of samples from VHHD locations (Figures 4A,D), while IgG and IL12 seemed to condition the position of the GN samples (Figures 4C,F) and Groα and IL7 that of the Peruvian samples in relation to the rest of the locations (Figures 4B,E). Median values for those immune factors exhibiting a cos^2^ > 0.2 are summarized as a heatmap in Figure 2B.

Furthermore, three major sample groups were detected; one included samples from SW and USC/USW, and located separately from all the rest of the samples. A second independent group of samples included those from GH, the only MHD country participating in this study. A third group contained the samples from PE and the LHD locations. Samples from Spain, a VHHD country, were positioned between the first and the third group of samples (Figures 4D–F).

**Figure 5:**
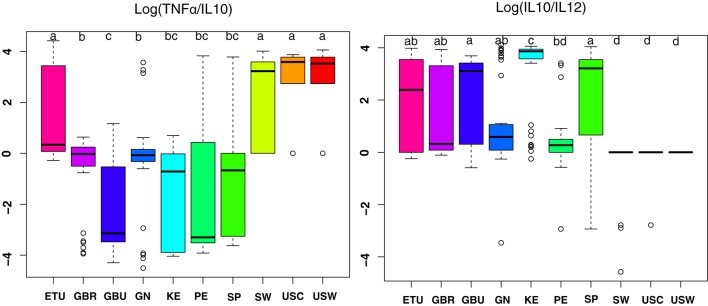
The ratio of TNFα/IL10 and IL10/IL12 in human milk ratios per geographical location. ETU, urban Ethiopia; GBR, rural Gambia; GBU, urban Gambia; GN, Ghana; KE, Kenya; PE, Peru; SP, Spain; SW, Sweden; USC, USA/California; USW, USA/Washington. Letters on top of each bar indicate significant differences (*p* < 0.05) on pairwise *post hoc* comparisons for each group (Kruskal–Wallis, Nemenyi *post hoc* test).

Since TNFα/IL10 and IL10/IL12 ratios have been associated with pro-inflammatory and anti-inflammatory states, respectively, they were further studied (Figure 5) (30). Overall, the highest anti-inflammatory ratios were found in samples from developing countries; among them, the KE samples exhibited the most pronounced ratio; and samples from USC, USW, and SW exhibited the lower ratios (*p* < 0.05). On the contrary, samples from VHHD countries, and particularly those from USC, USW, and SW, exhibited the highest TNFα to IL10 ratio as opposed to the GBU ones, which showed the lowest ratio.

### Potential Associations between Participants’ Data and Immunological Data

The effect of potential associations between the participants’ characteristics and the immunological variables was evaluated using a GLM. Those that were found to be statistically significant are summarized below, while *p*-values of the GLMs are shown in Table S2 in Supplementary Material. Among the participant characteristics, only delivery type, maternal age, time postpartum, and time since last feeding showed a significant covariation with location for some of the immunological factors evaluated. Groα covaried simultaneously with type of delivery, maternal age, and time postpartum. EGF and IL7 covaried with time from last feeding and type of delivery, respectively, while MIP1β and TNFα covaried with time postpartum.

## Discussion

Results from this study strongly support the concept that there is a common, but relatively small, “core” set of immunological soluble compounds present in mature milk produced by relatively healthy women, independent of their geographical location. We posit that these compounds are fundamentally important to infant (and/or mammary) health, regardless of contextual situation. Conversely, presence and/or concentration of many other immunological compounds vary geographically, which is in agreement with companion study conducted by us that focused on human milk oligosaccharides profiling within the same cohort (27). Perhaps these more “variable” substances are differently important, depending on location, environmental pathogen stress, hygiene, cultural norms, etc.

In the past, human milk was mainly considered as a source of nutrients for the developing infant. However, repetitive observations that breastfeeding significantly reduces morbidity and mortality rates associated with common diseases in both developed and developing countries have led to the recognition of additional key roles of human milk for infant health and homeostasis (31). From an immunological point of view, human milk contains a large number of immune elements (immune cells, cytokines, chemokines, growth factors, Ig, etc.) that provide passive protection during this period of host defense vulnerability (32). In addition, such elements contribute to the active maturation and shaping of the infant’s immune system and mucosal barriers (33, 34). Indeed, the lactating human mammary glands are a fundamentally relevant part of the MALT system during this crucial period of life (35). The mother’s mature immune system reacts efficiently to microorganisms and allergens to which she and her infant are exposed. Breastfeeding provides an ingenious immunologic integration between the mother’s and the infant’s immune systems (11).

Despite the recognized importance of milk’s immune factors (together with other bioactive factors) for the protection and development of the breastfed infant, studies on natural variations of the immunological composition of human milk among healthy women living in different geographical, dietary, and socioeconomical settings are scarce (25). To shed some light on this research gap, the present work investigated the presence and concentration of 23 soluble immune factors in a relatively large number of milk samples collected using matched protocols from healthy mothers living in high-, middle-, and low-income countries. Globally, our results provide considerable evidence that human milk immune factors exhibit high inter- and intra-variability across different populations, in agreement with previous observations (36, 37). Among the analyzed factors, only IgA, IgG, IgM, EGF, TGFβ2, IL7, IL8, Groα, and MIP1β were detected in all or most of the samples collected in each population at variable, yet biologically relevant, concentrations. TGFβ2, EGF, Groα, and IL8 were also detected in all the milk samples in previous studies (36, 37). Therefore, this specific set of compounds might be considered as the “core” soluble immune factors in milk produced by healthy women worldwide. Each of these factors has key roles in the barrier and immunological functions of the breastfed infant. They might also be important in protecting the mammary gland from disease during lactation.

Passively acquired maternal antibodies are important for protection against some pathogens in the neonatal period and promote long-term intestinal homeostasis by regulating the GI microbiota and host gene expression (38). Secretory IgA (sIgA) is the predominant Ig class found in human milk, compensating for the IgA deficiency of the infant and strongly contributing to the prevention of infant respiratory and GI infectious diseases (11). Both human milk IgA and IgM are active against a wide spectrum of viruses, bacteria, protozoa, yeast, and molds, inhibiting pathogens colonization and invasion (15, 33). Immune exclusion of antigens is performed mainly by sIgA in cooperation with innate defenses, but secretory IgM is also very relevant for neonatal health, being required for inactivating some Gram-negative pathogens (34, 39). In addition, sIgA seem to exert a role in the regulation of the immune response to dietary antigens since some studies have described an inverse relation between milk IgA levels and the development of allergy (40, 41).

Similar to IgA, the amount and repertoire of IgG produced by infants are clearly deficient because antigen-exposed memory T cells have not yet been generated. Transplacental transfer of IgG only partially corrects this deficiency since passively acquired IgG decrease rapidly after birth. The infant begins to actively produce IgG on exposure to antigens, but the complete antibody response is not achieved until 4–5 years of age, making infants particularly sensitive to encapsulated organisms (34), thus highlighting the protective value of breastfeeding in relation to mucosal infections.

Cytokines, chemokines, and growth factors are pluripotent polypeptides that operate in networks and coordinate the development and functions of the immune system. In the past, the study of such soluble factors in human milk has been difficult because of their complexity, their relatively low concentrations, and the lack of specific procedures and reagents to quantify such agents in this biological fluid. However, the number of such compounds that have been detected in human milk is growing rapidly. Although the actual physiologic effects of each of these factors in the infant have not been elucidated completely, their presence seems to be extraordinarily relevant for infant and mammary health (42, 43). Therefore, there is a growing interest in their roles and complex interactions, not only among them but also with other immunological and defense factors present in milk and/or the infant GI tract (lysozyme, lactoferrin, HMO, mucins, functional lipids, antimicrobial peptides and proteins, polyamines, microorganisms, etc.) (44).

In agreement with the results of our work, previous studies have shown that presence of variable (but usually high) concentrations of TGF-β2 is a common feature of human milk under physiological conditions (45, 46). TGF-β is considered as a key immunomodulatory factor in human milk (47, 48), and its importance is highlighted by the fact that endogenous GI TGF-β synthesis is defective in the neonate (49). TGF-β is critical for oral tolerance induction and global regulation of intestinal immune responses after food ingestion (50, 51). Epidemiologic studies have shown a positive correlation between levels of TGF-β in human milk and protection against wheeze and atopic dermatitis in breastfed children (52, 53), while animal studies have demonstrated the ability of TGF-β to prevent allergy in allergic-prone rats (54) and intestinal mucosa inflammation (55). In addition, TGF-β2 specifically attenuates IL1β-induced inflammatory responses in the immature human intestine *via* an SMAD6- and ERK-dependent mechanism (56). More recently, it has been observed that TGF-β2 and endotoxin interact to regulate homeostasis *via* IL8 levels in the immature intestine (57).

Chemokines are well known for their classic leukocyte chemoattractant activity, which is critical for directing the immune response to sites of infection and injury (58). Our work suggests that chemokines Groα (or CXCL1), IL8 and, to a lesser extent MIP1β, are included in the immunological core of human milk. Groα plays a role in spinal cord development by inhibiting the migration of oligodendrocyte precursors (59). This chemokine decreased the severity of multiple sclerosis in a mouse model and may provide a neuroprotective function (60). Additionally, Groα is involved in some processes that are essential in early life, such as angiogenesis and wound healing (61, 62).

Previous work investigating the presence of some chemoattractant factors (IL8, RANTES, eotaxin, IL16, MIP1α) in human milk revealed that only IL8 was present in 100% of the samples (63). Therefore, this chemokine may be particularly relevant for the trafficking of leukocytes from maternal circulation to the mammary gland and into milk. The production of IL8 by neonatal cells is reduced compared with adult cells (64) but, as with other immune factors, this developmental delay may be compensated for by ingesting human milk. A recent study reported that IL8 levels decline with stage of lactation (65). This is in contrast with our data since the mean concentration of this chemokine in KE samples (median: 74-day postpartum) was greater than in SW samples (median: 42-day postpartum). This suggests that there may be other factors influencing or determining IL8 levels in human milk.

In relation to growth factors, EGF was present in all the samples analyzed in this study. EGF enhances proliferation and differentiation of epithelial cells in the GI tract (16) and has significant effects on healing of damaged mucosa after injury (66, 67). The major sources of EGF for the infant GI tract are human colostrum and mature milk (68, 69). EGF in human milk has a protective effect against neonatal intestinal diseases, such as necrotizing enterocolitis (NEC) (70). This EGF-mediated protection against NEC has been associated to the well-known role of this growth factor in altering the balance of pro-apoptotic and anti-apoptotic proteins (71). Oral administration of EGF to rats with NEC-like symptoms decreased intestinal permeability, increased mucin production by goblet cells, and improved intestinal structure (72). All these changes improved GI integrity and enhanced intestinal barrier function. EGF may also contribute to the increased thymus size of breastfed (compared to formula-fed) infants (73). This might lead to a more advanced T lymphocyte differentiation and maturation, and consequently reduced risk of self-induced autoimmune disease. In this context, levels of IL7, a cytokine recently described in human milk and a common feature of the samples analyzed in this study, may correlate with improved thymus function in children (74).

The remaining cytokines, chemokines, and growth factors were found at variable detection frequencies and concentrations depending not only on the locations but also from one mother to another. Therefore, they could be considered as the “variable” set of soluble immune factors in human milk. Such physiological changes in the profile of cytokines may reflect individual patterns in the immune system of the mammary gland or the evolving needs of the recipient infants (75).

Despite this otherwise expected variability, the immune profiles obtained in this study allowed the clustering of the samples into groups highly concordant with the geographical origin of the samples and/or the HDI of the corresponding locations. Globally, the profiles in developing locations were consistent with a greater immune response plasticity, capable to exert protection against a broad range of stimuli, as supported by the higher number of high co-occurrence factors, including immune modulators (IL10 and GCSF) and acute response mediators (IL1β and MCP1), and the higher anti-inflammatory IL10/IL12 ratio. In the frame of the “Hygiene Hypothesis” this may reflect a higher level of maternal exposure to microorganisms and other antigens, which have been traditionally associated with developing countries. On the contrary, the immune profile of samples from developed locations was characterized by the low number of detected immune factors and the higher levels of IgA and EGF. This is consistent with a dominance of B cell activity as opposed to T cell-mediated immunity, suggesting a role for some practices that are generalized in Western lifestyle countries (e.g., hygienic birth practices, reduced contact with animals, safe food, water sanitation, sewage treatment, vaccination, use of antibiotic, anti-inflammatory or corticoids drugs, etc.). In this context, it is worth noting that the two populations from Gambia included in this study, with the same ethnic origin but living in two different environmental settings (rural and urban), exhibited significant differences in some immune factors. Additional studies will be needed to understand the drivers of these differences. Nonetheless, globally, the detection frequency and the concentration of pro-inflammatory TNFα and the detection frequency of some factors related to acquired immunity (IL17 and IL5), known to develop through life as a result of antigenic exposures and, therefore, tightly related to environmental pressures, were greater among the women living in the rural environment. In addition GCSF, known to participate in dendritic cells maturation and macrophage activity, exhibited detection frequencies much higher in the rural (75%) than in the urban population (25%). It is also worth remarking that TNFα to IL10 ratios were generally higher in the rural population. The factors that may explain, at least partly, the differences between these two genetically related populations include (a) less contact with animals, which is associated to a reduced contact with microorganisms (76) and (b) a higher BMI in GBU women, a factor generally associated with a state of inflammation and a negative impact on host immunity (77).

In this study, significant differences were found in several demographic, anthropometric, and mother/infant health-related factors but, as determined by GLMs, few significant associations could be established between these factors and immune profiles. This is presumably due to the high heterogeneity of the studied populations, which makes necessary a very high number of participants in order to elucidate the influence of different host and environmental factors on the immune composition of human milk. Recent work highlighted that, even within more homogeneous populations, the high variability in both milk immune profiles and environmental characteristics of the subjects might hinder the establishment of robust correlations (25). In fact, our results showed the existence of a significant covariation between some of the immune factors and certain demographic characteristics, such as including postpartum time or maternal age (Table S2 in Supplementary Material); unfortunately, no clear patterns could be identified, probably due to the high degree of variation in the demographic characteristics among the different populations analyzed in this study. Nonetheless, multivariate analyses revealed that samples from similar socioeconomic environments tend to cluster together, suggesting that common pressures might drive the presence of specific immune factors in human milk which, eventually, might be evolutionary fixed (78).

As stated above, several factors have been suggested to affect the immune composition of human milk, including the health status of the mother-infant dyad (18, 20, 79–81). Increased exposure to pathogens, as those occurring in clinical and subclinical mastitis (82, 83) and during infectious disease of the breastfed infant (84–86), might relate to increased pro-inflammatory factors in human milk. Although all the subjects recruited for the study were self-identified as healthy, the study could be biased as we cannot exclude the possibility that some of the participants were incubating or suffered from non-diagnosed or subclinical infections.

Time postpartum was another characteristic exhibiting variation among some of the population groups analyzed in this work. Some studies have reported that the immunological composition of human milk changes over lactation (87, 88). Although the most dramatic changes occur in the transition from colostrum to mature milk, this variable may be responsible, at least partly, for some of the differences observed in mature milk. In fact, postpartum time was the demographic characteristic that significantly covaried with a higher number of immune factors (Groα, MIP1β, and TNFα) in this work. Variation in human milk immune factors with lactational age might reflect fine-tuning of milk bioactive compounds according to the changing infant needs.

Dietary and nutritional differences among the compared populations might also account for, at least, a part of the variability observed among the human milk immune factors analyzed in this study. On the one hand, recruited women might suffer from food limitation and nutritional deficiencies/excesses that were not taken into consideration in this study. Therefore, it would be highly recommended to include a detailed nutritional assessment in further studies addressing human milk composition. On the other hand, certain foods or supplements, such as fish oil (17), black currant seed oil (80), or probiotic bacteria, more commonly used in certain population groups (79, 89–91), might modify the immunological composition of human milk. In our study, declaration of consumption of probiotic supplements and/or fermented foods by the participants significantly varied among locations, with the highest consumption rates (30%) in VHHD countries. However, since studies with different probiotic strains administered either during pregnancy and/or lactation led to different milk immune outcomes (91–93), no conclusion can be made regarding the impact of probiotic intake on the data of our study.

In summary, our study provides evidence that there is no one-size-fits-all immunological composition of milk produced by healthy women. Instead, there is substantial variation within and, particularly among, human subpopulations in this regard. Nonetheless, our data suggest the existence of a common “core” set of Ig, cytokines, chemokines, and growth factors that are present in mature milk produced by all women, independent of their origin. Other “variable” components may be differentially important to infant health due to location, culture, breastfeeding norms, etc. Additional studies are required to further elucidate relationships among specific host, geographical, environmental, lifestyle, and health variables and the immune composition of colostrum, transient milk, and mature human milk.

As a global conclusion, human milk is a complex and dynamic fluid that provides nutrients, antigens, passive immunity, GI growth factors, and bioactive compounds that can actively shape and educate the infant immune system. The immunological potential of milk differs from one mother to another and likely depends on a mother’s exposure to antigens, her immune responses to them, and the dose in milk of the wide array of cells and compounds with immunological activities. A better understanding of how the levels of these compounds in milk are controlled and the identification of the key promoters of anti-infectious and tolerance-induction properties in neonates should help in the establishment of new strategies to prevent infant diseases (45, 46). This clearly represents a major challenge in the frontiers of immunology.

## Ethics Statement

This study was carried out in accordance with the recommendations of the Washington State University Institutional Review Board guidelines. Ethics approvals were obtained for all procedures from each participating institution, with overarching approval from the Washington State University Institutional Review Board (#13264). After being translated from English (when needed), informed, verbal, or written consent (depending on locale and the subject’s literacy level) was acquired from each participating subject.

## Author Contributions

LR, IE-M, CG-C, SM, MKM, CM, MAM, JW, EK-M, EK, SM, LK, GO, KL, and KF conducted the research; MKM, CM, MAM, JF, DS, SEM, LK, GO, JR, RP, and LB designed the research; LR, IE-M, CG-C, and JR wrote the manuscript; LR, IE-M, and JR had primary responsibility for the final content of the manuscript; LR, IE-M, and CG-C analyzed the data; and all authors read and approved the final manuscript. None of the authors reported a conflict of interest related to the study.

## Conflict of Interest Statement

The authors declare that the research was conducted in the absence of any commercial or financial relationships that could be construed as a potential conflict of interest.

## Abbreviations

BMI, body mass index; ETU, urban Ethiopia; GBR, rural Gambia; GBU, urban Gambia; GCSF, granulocyte colony-stimulating factor; GMCSF, granulocyte–macrophage colony-stimulating factor; GI, gastrointestinal; GN, Ghana; GLM, general linear model; Groα, growth-related oncogene-α; HDI, human development index; HHD, high human development; IPAb, intrapartum antibiotherapy; IFNγ, interferon-γ; Ig, immunoglobulin; IL, interleukin; KE, Kenya; LHD, low human development; MALT, mucosa-associated lymphoid tissue; MCP1, macrophage–monocyte chemoattractant protein-1; MHD, medium human development; MIP1β, macrophage inflammatory protein-1β; PCA, principal component analysis; PE, Peru; sIg, secretory immunoglobulin; SP, Spain; SW, Sweden; TGFβ2, transforming growth factor-β2; TNFα, tumor necrosis factor-α; USW, USA/Washington; USC, USA/California (Hispanic); VHHD, very-high human development.
