# Supplementary material for: What’s Normal? Immune Profiling of Human Milk from Healthy Women Living in Different Geographical and Socioeconomic Settings
Source: Front Immunol. 2017 Jun 30;8:696. doi: 10.3389/fimmu.2017.00696 (PMC5492702; doi:10.3389/fimmu.2017.00696)

Supplementary Material

What's normal? Immune profiling of human milk from healthy women living in different geographical and socio-economic settings

Lorena Ruiz,[1*&] PhD, Irene Espinosa-Martos,[1,2*&] PhD, Cristina García,[1] MSc, Susana Manzano,[1] PhD, Michelle K. McGuire,[3,4] PhD, Courtney L. Meehan,[5] PhD, Mark A. McGuire,[6] PhD, Janet E. Williams,[6] PhD, James Foster,[7] PhD, Daniel W. Sellen,[8] PhD, Elizabeth W. Kamau-Mbuthia,[9] PhD, Egidioh W. Kamundia,[9] PhD, Samwel Mbugua,[9] PhD, Sophie E. Moore,[10,11] PhD, Linda J. Kvist,[12] PhD; Gloria E. Otoo,[13] PhD; Kimberly A. Lackey,[3] BS, Katherine Flores[5], MA, Rossina G. Pareja,[14] MS, Lars Bode,[15] PhD, and Juan M. Rodríguez,[1*] PhD

*To whom correspondence should be addressed:

Lorena Ruiz: lorena.ruiz@ipla.csic.es

Irene Espinosa: irenee70@gmail.com

Juan Miguel Rodriguez: jmrodrig@vet.ucm.es

Supplementary Figure S1

Heatmap representing the levels of the 16 immune factors contributing the most to the samples separation according to the multivariate analysis conducted in this work (cos2 > 0.2). Rows are mean centered; upper bar indicates the sampling countries/locations; the dendrogram has been drawn based on Euclidean distances.

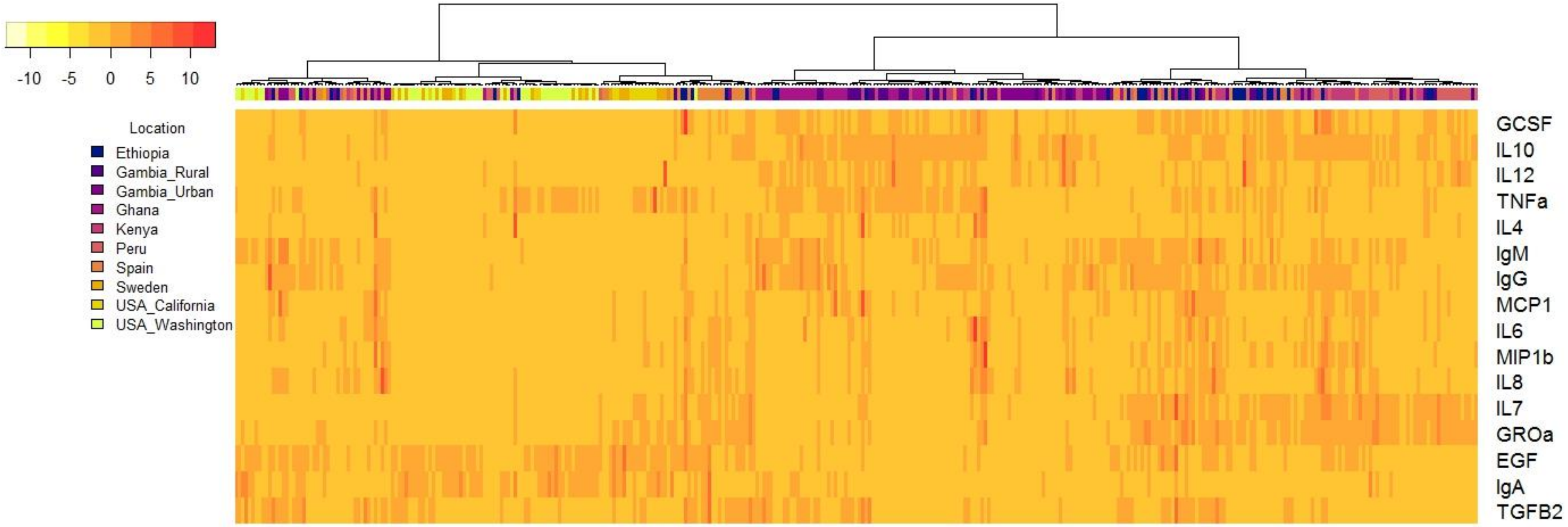

Supplementary Figure S2

Heatmap representing the levels of the 16 immune factors contributing the most to the samples separation according to the multivariate analysis conducted in this work (cos2 > 0.2). Rows are mean centered, upper bar indicates the human development index classification of the location where the corresponding sample was collected from; the dendrogram has been drawn based on Euclidean distances. LHD: low human development; MHD: medium human development; HHD: high human development; VHHD: very high human development.

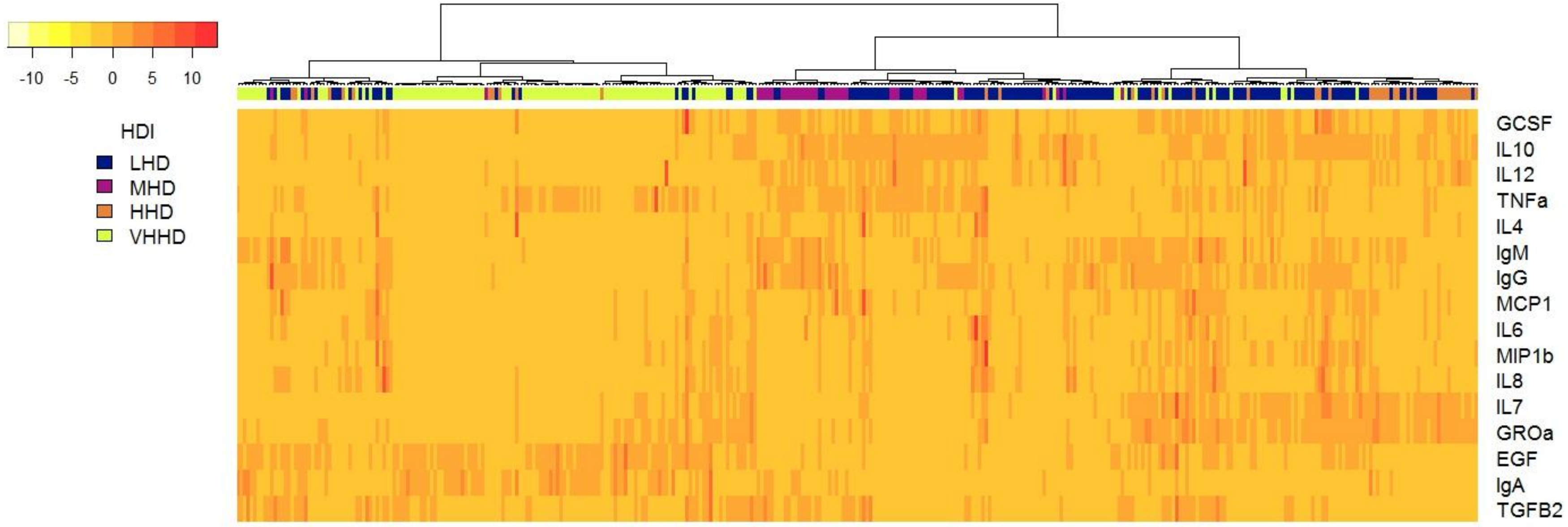

Supplementary Figure S3.

Circular dendrogram including 365 of the analyzed samples, drawn on the basis of Kendall correlation coefficient matrix. Samples are color-coded according to the country where they were collected.

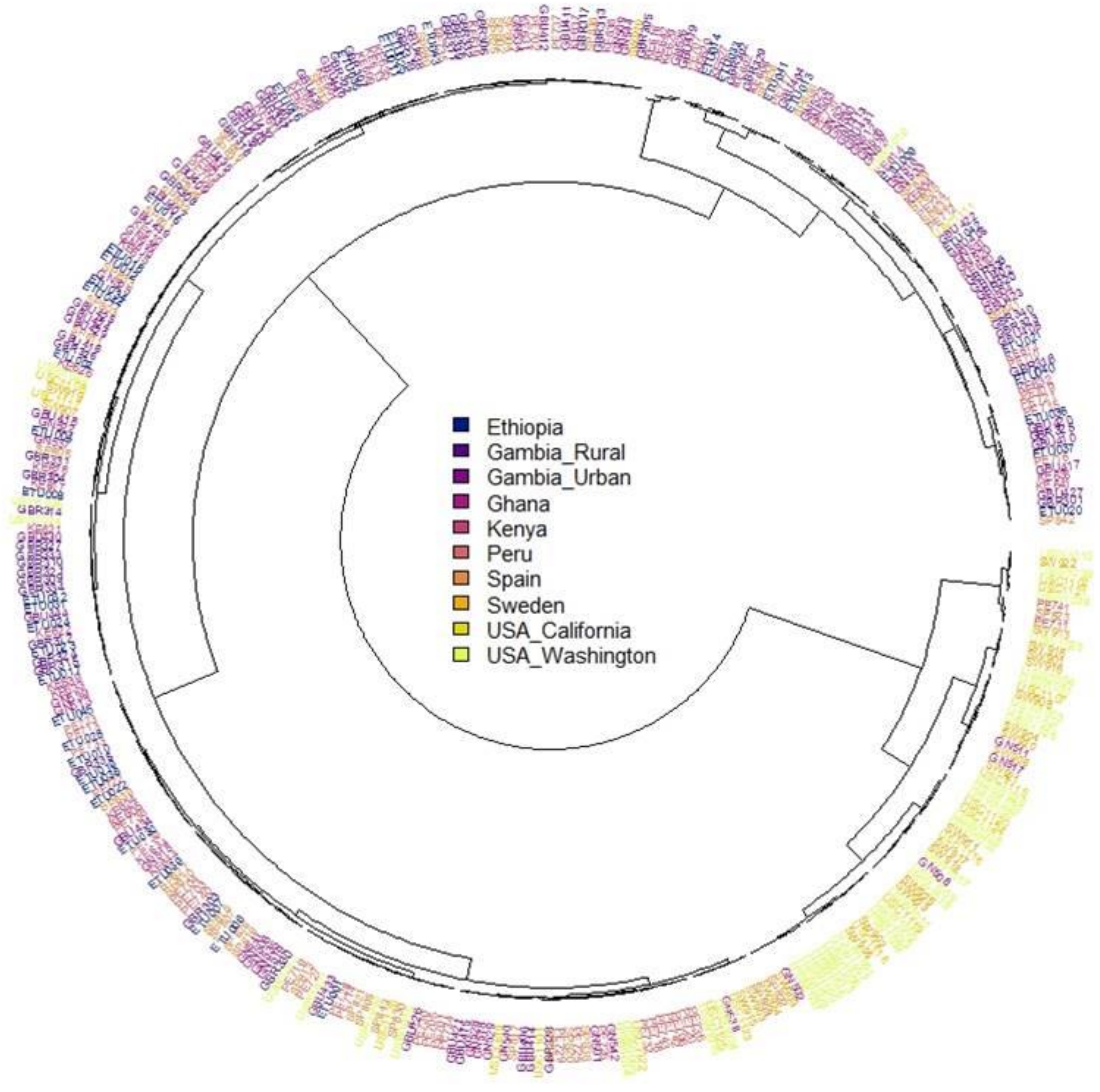

Supplement: Supplementary file 1 [file Image_1.PDF]
