# Supplementary material for: What’s Normal? Immune Profiling of Human Milk from Healthy Women Living in Different Geographical and Socioeconomic Settings
Source: Front Immunol. 2017 Jun 30;8:696. doi: 10.3389/fimmu.2017.00696 (PMC5492702; doi:10.3389/fimmu.2017.00696)
Supplement: Supplementary file 2 [file Table_1.DOC]

Supplementary Table 1

**What’s normal? Immune profiling of human milk from healthy women living in different geographical and socio-economic settings**

Lorena Ruiz,^1*&^ PhD, Irene Espinosa-Martos,^1,2*&^ PhD, Cristina García,^1^ MSc, Susana Manzano,^1^ PhD, Michelle K. McGuire,^3,4^ PhD, Courtney L. Meehan,^5^ PhD, Mark A. McGuire,^6^ PhD, Janet E. Williams,^6^ PhD, James Foster,^7^ PhD, Daniel W. Sellen,^8^ PhD, Elizabeth W. Kamau-Mbuthia,^9^ PhD, Egidioh W. Kamundia,^9^ PhD, Samwel Mbugua,^9^ PhD, Sophie E. Moore,^10,11^ PhD, Linda J. Kvist,^12^ PhD; Gloria E. Otoo,^13^ PhD; Kimberly A. Lackey,^3^ BS, Katherine Flores^5^, MA, Rossina G. Pareja,^14^ MS, Lars Bode,^15^ PhD, and Juan M. Rodríguez,^1*^ PhD

*To whom correspondence should be addressed:

Lorena Ruiz: [lorena.ruiz@ipla.csic.es](mailto:lorena.ruiz@ipla.csic.es)

Irene Espinosa: [irenee70@gmail.com](mailto:irenee70@gmail.com)

Juan Miguel Rodriguez: jmrodrig@vet.ucm.es

**Supplementary Table 1.**

**Table S1**. Low limit of quantification (LLOQ) for every analyte in human milk. LLOQ values are expressed as ng/L for cytokines, mg/L for immunoglobulins, and μg/L for EGF, TGFβ2 and Groα.

| **Functional classification of quantified compounds** | **Analyte** | **LLOQ (units)** |
| --- | --- | --- |
| **Innate immunity** | IL1β | 0.027 |
|  | IL6 | 0.082 |
|  | IL12 | 0.131 |
|  | INFγ | 0.659 |
|  | TNFα | 0.709 |
| **Acquired immunity** | IL2 | 2.179 |
|  | IL4 | 0.139 |
|  | IL10 | 0.215 |
|  | IL13 | 0.081 |
|  | IL17 | 1.571 |
|  | IL5 | 0.213 |
|  | IL7 | 0.512 |
|  | IgA | 2.03E-04 |
|  | IgM | 7.61E-04 |
|  | IgG | 2.70E-03 |
|  | TGFβ2 | 1.24E-03 |
| **Chemokines** | GCSF | 0.388 |
|  | GMCSF | 0.537 |
|  | EGF | 2.70E-05 |
| **Growth factors** | IL8 | 0.418 |
|  | Groα | 2.289 |
|  | MCP1 | 3.219 |
|  | MIP1β | 0.216 |
