# Supplementary material for: What’s Normal? Immune Profiling of Human Milk from Healthy Women Living in Different Geographical and Socioeconomic Settings
Source: Front Immunol. 2017 Jun 30;8:696. doi: 10.3389/fimmu.2017.00696 (PMC5492702; doi:10.3389/fimmu.2017.00696)
Supplement: Supplementary file 3 [file Table_2.DOC]

Supplementary Table 2

**What’s normal? Immune profiling of human milk from healthy women living in different geographical and socio-economic settings**

Lorena Ruiz,^1*&^ PhD, Irene Espinosa-Martos,^1,2*&^ PhD, Cristina García,^1^ MSc, Susana Manzano,^1^ PhD, Michelle K. McGuire,^3,4^ PhD, Courtney L. Meehan,^5^ PhD, Mark A. McGuire,^6^ PhD, Janet E. Williams,^6^ PhD, James Foster,^7^ PhD, Daniel W. Sellen,^8^ PhD, Elizabeth W. Kamau-Mbuthia,^9^ PhD, Egidioh W. Kamundia,^9^ PhD, Samwel Mbugua,^9^ PhD, Sophie E. Moore,^10,11^ PhD, Linda J. Kvist,^12^ PhD; Gloria E. Otoo,^13^ PhD; Kimberly A. Lackey,^3^ BS, Katherine Flores^5^, MA, Rossina G. Pareja,^14^ MS, Lars Bode,^15^ PhD, and Juan M. Rodríguez,^1*^ PhD

*To whom correspondence should be addressed:

Lorena Ruiz: [lorena.ruiz@ipla.csic.es](mailto:lorena.ruiz@ipla.csic.es)

Irene Espinosa: [irenee70@gmail.com](mailto:irenee70@gmail.com)

Juan Miguel Rodriguez: jmrodrig@vet.ucm.es

**Supplementary Table S2.**

**Table S2.** Statistical significance of generalized linear models. Only those immune factors that evidenced significant co-variation with, at least, one of the demographic/medical variables studied are indicated.

|  | **Demographic/medical variables*** | | | |
| --- | --- | --- | --- | --- |
| **Factor** | **Delivery** | **MAge** | **Ppdays** | **Tlastfeed** |
| EGF | 0.260 | 0.626 | 0.474 | 0.007 |
| GROα | 0.001 | <0.001 | 0.044 | 0.824 |
| IL7 | 0.019 | 0.424 | 0.838 | 0.924 |
| MIP1β | 0.634 | 0.727 | <0.001 | 0.713 |
| TNFα | 0.234 | 0.595 | 0.007 | 0.052 |
| *Delivery, type of delivery (c-section vs. vaginal); MAge, maternal age;  Ppdays, days postpartum;Tlastfeed, time since last feeding. | | | | |
